# A survey of contemporary usage of epicardial pacing wires among UK cardiothoracic surgeons: A call for a more conservative approach

**DOI:** 10.1186/1749-8090-10-S1-A342

**Published:** 2015-12-16

**Authors:** Vivek Srivastava, Pankaj Kumar Mishra, Enoch Akowuah, Joel Dunning, Jonathan Ferguson, Andrew Goodwin, Simon Kendall, Andrew Owens, Ralph White

**Affiliations:** 1 Department of Cardiothoracic Surgery, James Cook University Hospital, Middlesbrough, TS4 3BW, UK

## Background/Introduction

To determine current practice regarding the use of epicardial pacing wires by cardiothoracic surgeons in the U.K.

## Aims/Objectives

To determine current practice regarding the use of epicardial pacing wires by cardiothoracic surgeons in the U.K.

## Method

An internet-based survey was distributed via email to all U.K. cardiac and cardiothoracic surgeons. The questionnaire consisted of 18 questions regarding use and management of epicardial pacing wires.

## Results

Of 282 questionnaires, 126 responses were received (response rate 44.7%). Around two thirds (68.3%) of respondents routinely used epicardial wires for isolated coronary artery bypass grafts (CABG). Both atrial and ventricular wires were favoured for valve cases: isolated aortic valve(60.3% respondents), isolated mitral valve(63.5%), multiple valves(70%), CABG & valve(63.5%), redo valve(67.5%). The main reasons quoted for not using pacing wires: perception as an unnecessary procedure(22.2%), risk of bleeding(25.4%) and potential for delayed discharge(17.5%). Around half (54%) of surgeons reported practising minimally invasive techniques and 36.8% of these modified pacing wire usage. Two-thirds of surgeons accepted an INR of <2.5 for removal of pacing wires with another 24.6% accepting an INR <3.0 (>91% overall). Seventy percent would not remove pacing wires outside daytime hours although 54% removed them over weekends and holidays. Postoperative day 3 or 4 was the most common day for removal. Forty-five percent of respondent surgeons were comfortable discharging patients the day the wires were removed.

## Discussion/Conclusion

Results show considerable variation in practice. Modifications based on peer practice could potentially save bed-days (by increasing pacing wire removal over weekends and out-of-hours and same-day discharge), reduce costs (clarifying indications and reducing routine use) and reduce risk of bleeding (by standardising safe level of anticoagulation).

